# Costs and staffing resource requirements for adaptive clinical trials: quantitative and qualitative results from the Costing Adaptive Trials project

**DOI:** 10.1186/s12916-021-02124-z

**Published:** 2021-10-26

**Authors:** Nina Wilson, Katie Biggs, Sarah Bowden, Julia Brown, Munyaradzi Dimairo, Laura Flight, Jamie Hall, Anna Hockaday, Thomas Jaki, Rachel Lowe, Caroline Murphy, Philip Pallmann, Mark A. Pilling, Claire Snowdon, Matthew R. Sydes, Sofía S. Villar, Christopher J. Weir, Jessica Welburn, Christina Yap, Rebecca Maier, Helen Hancock, James M. S. Wason

**Affiliations:** 1 Population Health Sciences Institute, Newcastle University, Baddiley-Clark Building, Richardson Road, Newcastle upon Tyne, NE2 4AX UK; 2 School of Health and Related Research (ScHARR), University of Sheffield, Sheffield, UK; 3 Cancer Research UK Clinical Trials Unit (CRCTU), University of Birmingham, Birmingham, UK; 4 Leeds Institute of Clinical Trials Research, University of Leeds, Leeds, UK; 5 MRC Biostatistics Unit, University of Cambridge, Cambridge, UK; 6 Department of Mathematics and Statistics, Lancaster University, Lancaster, UK; 7 Centre for Trials Research, Cardiff University, Cardiff, UK; 8 King’s College Trials Unit, King’s College London, London, UK; 9 Department of Public Health and Primary Care, University of Cambridge, Cambridge, UK; 10 The Institute of Cancer Research Clinical Trials & Statistics Unit, London, UK; 11 MRC Clinical Trials Unit at UCL, London, UK; 12 Edinburgh Clinical Trials Unit, Usher Institute, University of Edinburgh, Edinburgh, UK; 13 Newcastle Clinical Trials Unit, Newcastle University, Newcastle upon Tyne, UK

**Keywords:** Adaptive designs, Adaptive clinical trials, Clinical trials, Efficiency, Resource requirements, Trial coordination

## Abstract

**Background:**

Adaptive designs offer great promise in improving the efficiency and patient-benefit of clinical trials. An important barrier to further increased use is a lack of understanding about which additional resources are required to conduct a high-quality adaptive clinical trial, compared to a traditional fixed design.

The Costing Adaptive Trials (CAT) project investigated which additional resources may be required to support adaptive trials.

**Methods:**

We conducted a mock costing exercise amongst seven Clinical Trials Units (CTUs) in the UK. Five scenarios were developed, derived from funded clinical trials, where a non-adaptive version and an adaptive version were described. Each scenario represented a different type of adaptive design.

CTU staff were asked to provide the costs and staff time they estimated would be needed to support the trial, categorised into specified areas (e.g. statistics, data management, trial management). This was calculated separately for the non-adaptive and adaptive version of the trial, allowing paired comparisons.

Interviews with 10 CTU staff who had completed the costing exercise were conducted by qualitative researchers to explore reasons for similarities and differences.

**Results:**

Estimated resources associated with conducting an adaptive trial were always (moderately) higher than for the non-adaptive equivalent. The median increase was between 2 and 4% for all scenarios, except for sample size re-estimation which was 26.5% (as the adaptive design could lead to a lengthened study period). The highest increase was for statistical staff, with lower increases for data management and trial management staff.

The percentage increase in resources varied across different CTUs. The interviews identified possible explanations for differences, including (1) experience in adaptive trials, (2) the complexity of the non-adaptive and adaptive design, and (3) the extent of non-trial specific core infrastructure funding the CTU had.

**Conclusions:**

This work sheds light on additional resources required to adequately support a high-quality adaptive trial. The percentage increase in costs for supporting an adaptive trial was generally modest and should not be a barrier to adaptive designs being cost-effective to use in practice.

Informed by the results of this research, guidance for investigators and funders will be developed on appropriately resourcing adaptive trials.

**Supplementary Information:**

The online version contains supplementary material available at 10.1186/s12916-021-02124-z.

## Background

Clinical trials are vital for demonstrating safety, efficacy, and effectiveness of interventions aimed at improving patient health. The cost of conducting trials is high and is increasing; it is a major factor behind the increasing costs of drug development [1] and evaluation of non-pharmacological interventions [2]. Because of this, there has been a big drive towards developing trial methods that can increase operational and statistical efficiency.

One important class of methods is adaptive trial designs [3]. An adaptive design, according to the adaptive CONSORT extension [4], ‘offers pre-planned opportunities to use accumulating trial data to modify aspects of an ongoing trial while preserving the validity and integrity of that trial’. There are many types of adaptive trial designs that can be used for different purposes. Generally, they have one or more of the following objectives compared to traditional trial designs: (1) improving the power of the trial, (2) reducing the average sample size used and trial duration for a target level of power, and (3) improving outcomes of patients who are enrolled on the trial [5].

The use of adaptive designs has been increasing in recent years [6–8] although it still remains low when compared to traditional trial designs. Some barriers to increased use have already been identified [9–11]). These include lack of awareness of their purpose and potential, expertise, training, and availability of easy-to-use software for implementation, alongside a paucity of experiences in their delivery. Although there is still more to do, these barriers are being steadily addressed, especially for less complex adaptive methods. Adaptive designs have been widely and successfully used in trials of COVID-19 prevention and treatment [12], which will likely lead to further demand for their use.

One important area that has received less attention relates to the cost and resource needed to implement adaptive designs, which generally require interim analyses to be conducted quickly and to a high level of quality. Additionally, trials using adaptive designs are usually more complex and may require more effort to set up, manage, monitor, analyse, and close.

The complexities associated with adaptive designs may mean they require additional resource to develop and conduct. This might be difficult to quantify upfront (e.g. when developing a grant application) without having substantial knowledge and experience of developing and running trials using an adaptive design from start to finish. This issue is further exacerbated by lack of transparent data about the costs of different aspects of running trials [13]. Previous research has been published on resource needs for trials generally [14–16], but none focused on additional requirements for adaptive designs. A need for further research and guidance on this issue was identified within the MRC-NIHR Trials Methodology Research Partnership Adaptive Designs Working Group. This led to an application for funding that formed a national project team to investigate the resource requirements for adaptive trials and develop best-practice guidance for various stakeholders.

The objectives were:

1. To estimate the perceived additional (financial and staffing) resources needed to conduct trials using adaptive designs through conducting a mock costing exercise of several types of trial scenarios
2. To investigate reasons for differences between non-adaptive and adaptive trials through qualitative research
3. To provide best-practice guidance on what additional resources should be included in funding applications for trials using adaptive designs

This paper presents the research results of the CAT project, including the mock costing exercise project and the qualitative research (objectives 1 and 2). A separate paper will focus on providing guidance that arose from the research (objective 3).

## Methods

### Development of mock costing scenarios

A subgroup of the CAT team developed five trial scenarios for different Clinical Trials Units (CTUs) to provide costs for. These five scenarios were based on real and funded trials, although with some changes made to avoid any comparison to the CTU costs of the original trial.

In each scenario, a non-adaptive version of the trial was provided, with a protocol synopsis containing a summary of the trial’s PICO (Participants, Intervention(s), Comparator, Outcomes). The scenario also outlined a summary of the adaptive design proposed, with the rationale and implications it would have on the design.

Table 1 provides an overview of the different scenarios. A full description of each scenario is provided in Additional file 1.

**Table 1:** Brief overview of each scenario used in the mock costing exercise

Scenario/non-adaptive design	Adaptive design and features
1. A two-arm parallel-group randomised controlled trial assessing the addition of biomarker-testing to an existing early warning score in the management of patients with suspected sepsis in the emergency department	Group-sequential design^a^ including a single interim analysis with futility stopping after half of patients have had primary outcome observed.
2. A phase 2b randomised dose-finding study of JAK1 inhibitor for patients with active rheumatoid arthritis	Adaptive dose-finding design that has a single interim analysis after half of patients have primary outcome observed. The dose allocation used in the second stage is set according to an optimal allocation from a three-parameter emax model fitted to stage 1 patient outcomes.
3. A multi-arm parallel-group phase 3 trial comparing regimens for treating intermediate and high-risk oropharyngeal cancer	Multi-arm multi-stage design with two interim analyses (2 years and 4 years into a 5-year recruitment period) that allows early stopping of experimental arms for lack of benefit. The trial continues to full enrolment unless all experimental arms stop early.
4. A multi-arm parallel-group trial assessing clinical efficacy and cost-effectiveness of earlier treatment of ovarian hyper-stimulation syndrome (OHSS)	Adaptive umbrella design, allowing early stopping of arms within the two patient subgroups. In the early OHSS subgroup, a MAMS design is used with one interim analysis allowing stopping for lack of benefit; in the late OHSS subgroup, a group-sequential design with early stopping for lack of benefit is used.
5. Randomised two-arm parallel-group trial of the efficacy of nicotinic acid derivative (NAD) for treatment of fatigue in mitochondrial disease	Sample size re-assessment design that will use blinded estimate of the pooled standard deviation to re-estimate the sample size required. If this is above a specified level, the trial will stop early for futility.

^a^Group-sequential designs are not always considered an example of an adaptive design but were included in the definition within this project as they also involve a pre-specified interim analysis of outcome data

### Resource and cost data requested

A spreadsheet (Additional file 2) was provided to CTUs which asked for information on the resources required to undertake the costing of each scenario. This was split into the following: (1) pre-award the resources needed to develop the application to the point of the grant starting, (2) post-award staff resources that would be required to deliver the trial, and (3) post-award non-staff costs required to deliver the trial once the grant had started. Typically, pre-award resource needs are covered by the institution (e.g. using existing core infrastructure funding), with post-award costs mostly covered by the grant funder.

For staff resource, information was requested on staff type, co-applicant status, grade, contract type, whether the post was underpinned by central funding, the percentage of full-time equivalent (FTE) the staff member would be working on the project (either averaged over the length of the trial, or at different levels during the trial), total months on trial, total salary cost (including direct employment-related costs e.g. employer taxes and pension contributions), full economic costs (FEC) charged, and any additional indirect employment-related costs (e.g. recruitment costs). For non-staff resource, suggested cost items were listed including stationery, travel (trial oversight committees, site initiation visits, monitoring, closedown of centres), project meeting costs (launch and investigator meetings), teleconferencing fees, clinical data management and randomisation system fees, Medicines and Healthcare products Regulatory Agency (MHRA) fees (including amendments), computing costs (including specialist statistical software), staff training, patient and public involvement (PPI), dissemination (including open-access publication costs), data sharing, and post project costs (e.g. data archiving and anonymisation). Each CTU could also add any additional costs not suggested.

Staff resource and non-staff costs were collected for both the non-adaptive and adaptive versions of each scenario. Guidance was given to each participating CTU as comments within the spreadsheet and a separate guidance document (see Additional file 1). If CTU staff undertaking the costing exercise had queries, they were offered the opportunity to email the subgroup of the CAT team.

After receiving the completed costings, any queries were emailed directly to the person who completed them at the participating CTU. The majority of these queries were resolved; for the very few minor unresolved queries, assumptions were made based on the other information provided by that CTU.

### Participating Clinical Trials Units

In the initial grant application, some of the co-applicants were associated with CTUs. Additional CTUs were invited through an email sent to Directors of all 53 UK Clinical Research Collaboration (UKCRC)-registered CTUs, describing the project, in November 2019. All materials required for conducting the costing were sent out in January 2020, with a request to send back completed costings by March 2020.

### Analysis methods

Due to the large variation in the grades of staff at different institutions, it was decided to summarise requested staff resource using the number of ‘FTE-years’ without reference to grade or cost. The FTE-year for a staff post is the number of years that a post is funded on the grant multiplied by the average percentage of FTE. As an example, a staff member funded at 50% FTE for 4 years would contribute 2 FTE-years to the total staff resource requested.

FTEs were analysed as total, as well as by categories of staff by type. The main categories were statistics, data management, and trial management. Other categories included programming, administration, quality assurance, senior management/operations, data entry, and researcher; however, these were inconsistently used across CTUs and have not been included as individual staff groups in this manuscript (although they do contribute to the total resource calculated). Non-staff costs are presented as a total.

Staff FTE-years and non-staff costs are presented as a percentage change between the non-adaptive and adaptive design, and as a relative change for the adaptive design compared to the median non-adaptive design figure, for each scenario and CTU. This was after discussion with the project group and was implemented to ensure anonymity of the CTUs involved and due to commercial confidentiality. Statistical analysis was descriptive and the percentage change data was summarised as a median and range. Relative change for each scenario was summarised using spaghetti plots. A spaghetti plot has a separate line for each individual CTU that returned a costing for the scenario, linking the estimated resources for the non-adaptive and adaptive version of the scenario. Plots were created using the ggplot2 package [17] in R [18].

### Qualitative methods

Participants who undertook the costing exercise were invited to interview and all were interviewed via videoconference and audio recorded. A semi-structured topic guide was developed from previous work on costing trials [14] and at an investigators’ meeting in July 2020 following the costing exercise. This included questions relating to the differences across CTUs and explored local costing procedures and reasons for differences in costs between the adaptive and non-adaptive scenarios.

Qualitative interviews were transcribed verbatim and coded in NVivo (Windows, version 1.3). The National Centre for Social Research ‘Framework’ approach [19] (familiarisation, identifying a thematic framework, indexing, charting, and mapping and interpretation) was used for analysis. Themes were derived inductively from reading the transcripts.

The results were presented and discussed at an investigators’ meeting in January 2021.

### Funding and ethical approval

CAT was funded by the NIHR Efficient Studies Funding panel (Reference: NIHR130351). The funder played no role in the design, conduct, or reporting of the research. The project received an ethical waiver by Newcastle University’s ethics committee due to it not involving patients. Consent was obtained for all participants who took part in the interviews.

## Results

### Mock costing exercise results

A total of 10 CTUs expressed willingness to take part in the mock costing exercise. Of these, seven contributed at least one costing, with three providing a costing for all five scenarios; uptake and drop out was impacted by the COVID-19 pandemic, which substantially increased workload within CTUs from March 2020.

A costing for both the non-adaptive and adaptive versions of the scenario was always given. Each scenario had costings from at least five CTUs (Table 2).

**Table 2:** Summary of which CTU completed the costing exercise for each scenario

		Scenario				
		1. Group sequential design	2. Phase 2b dose response	3. Phase 3 MAMS	4. Umbrella study	5. Sample size re-estimation
**CTU**	**1**	✓	✓	✓	✓	✓
	**2**	✓	✓	✓		✓
	**3**				✓	
	**4**	✓	✓	✓		✓
	**5**	✓	✓	✓	✓	✓
	**6**	✓	✓	✓	✓	✓
	**7**	✓	✓		✓	

*CTU* clinical trials unit, *MAMS* multi-arm multi-stage; each CTU has been allocated an anonymous number

**Fig. 1:**
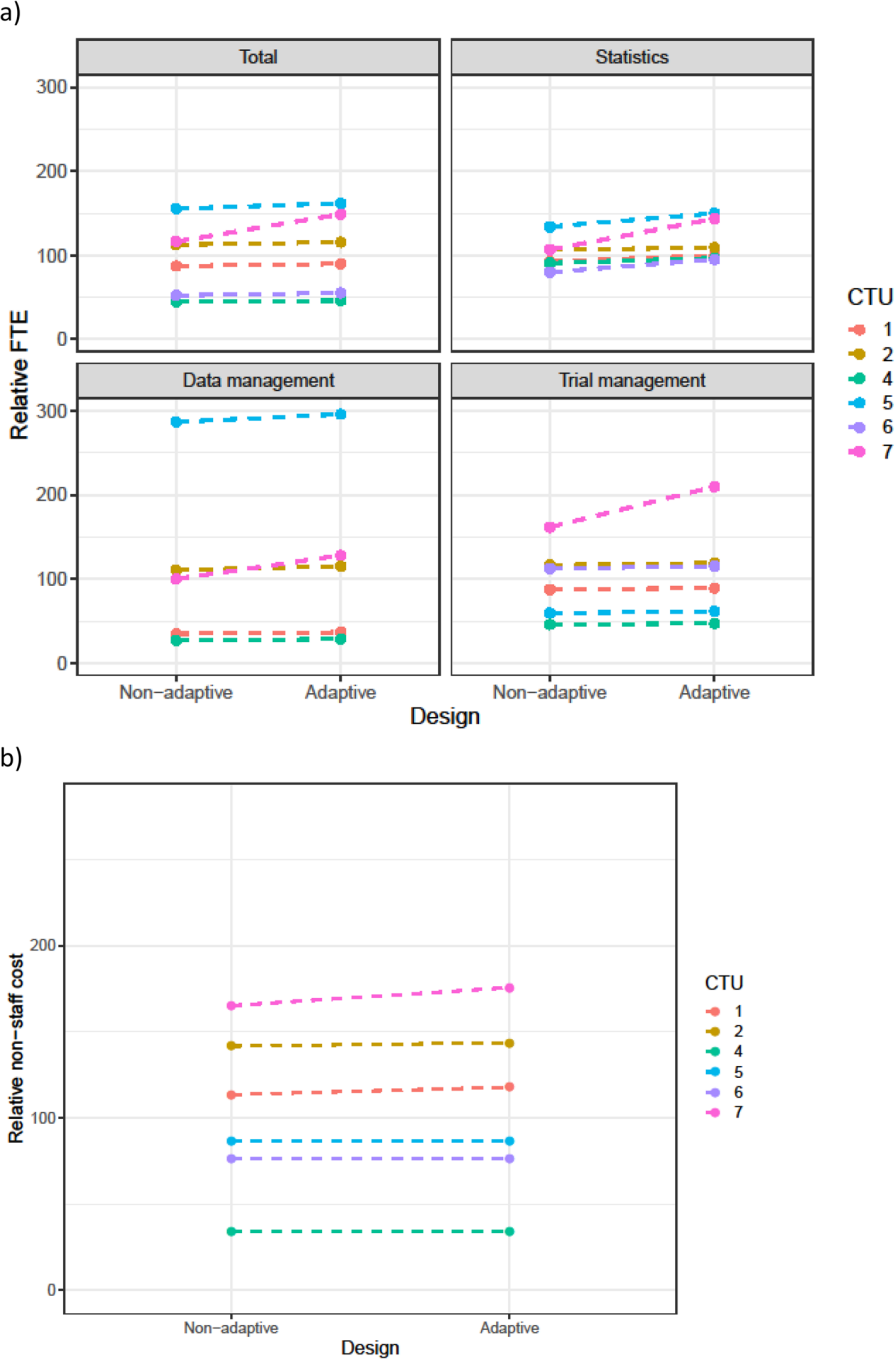
**a** FTE-years relative to non-adaptive median (set to 100) for scenario 1. **b** Total non-staff cost relative to non-adaptive median (set to 100) for scenario 1

**Fig. 2:**
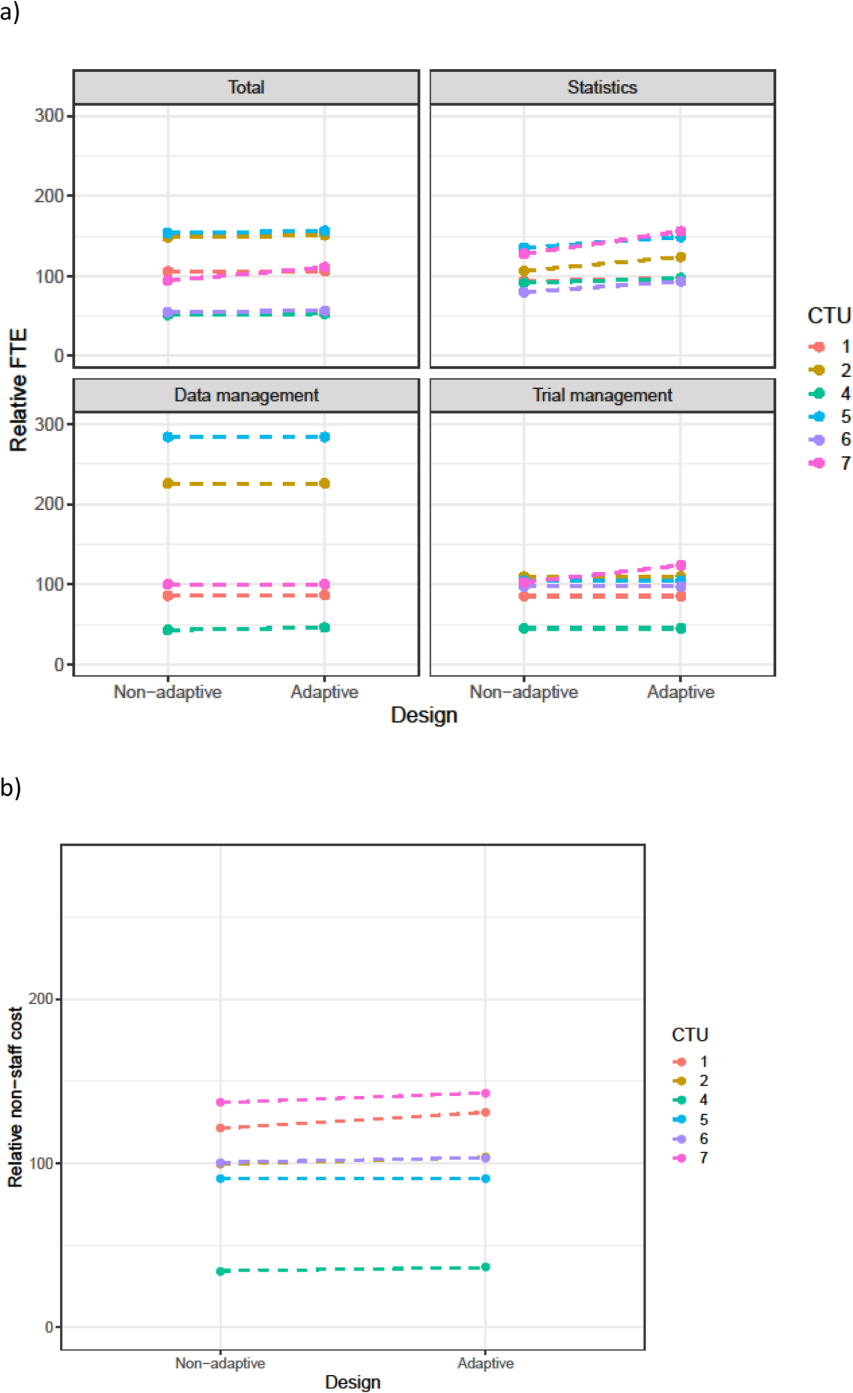
**a** FTE-years relative to non-adaptive median (set to 100) for scenario 2. **b** Total non-staff cost relative to non-adaptive median (set to 100) for scenario 2

**Fig. 3:**
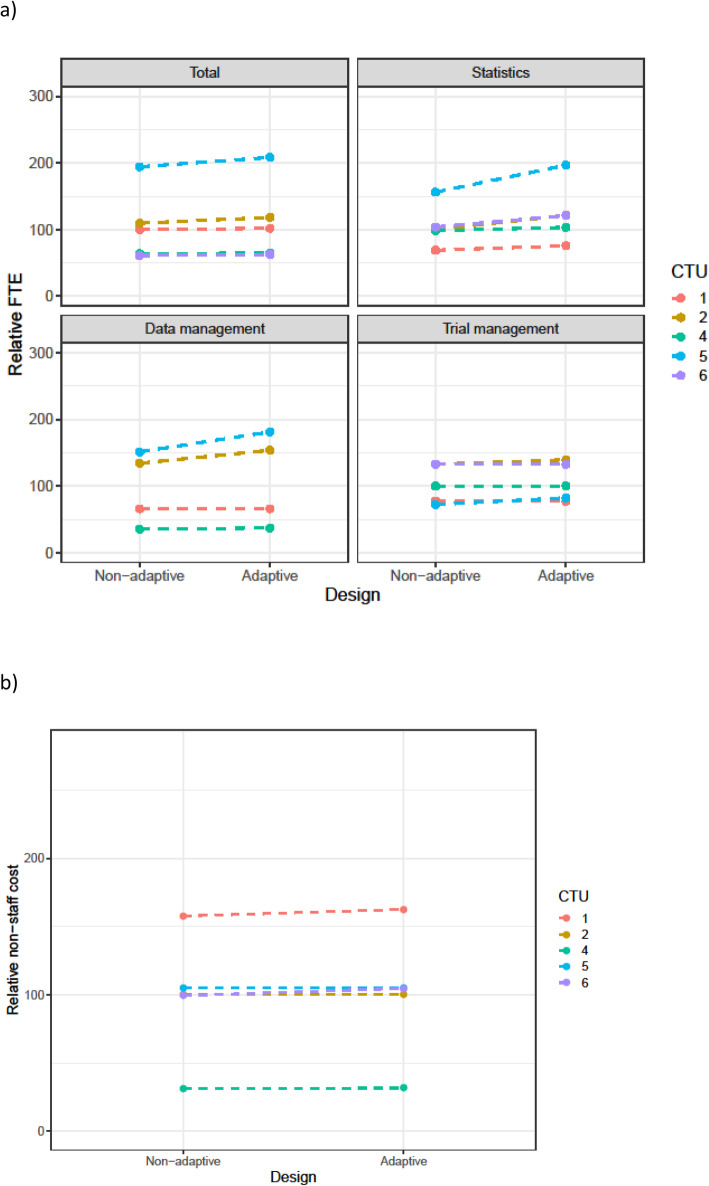
**a** FTE-years relative to non-adaptive median (set to 100) for scenario 3. **b** Total non-staff cost relative to non-adaptive median (set to 100) for scenario 3

**Fig. 4:**
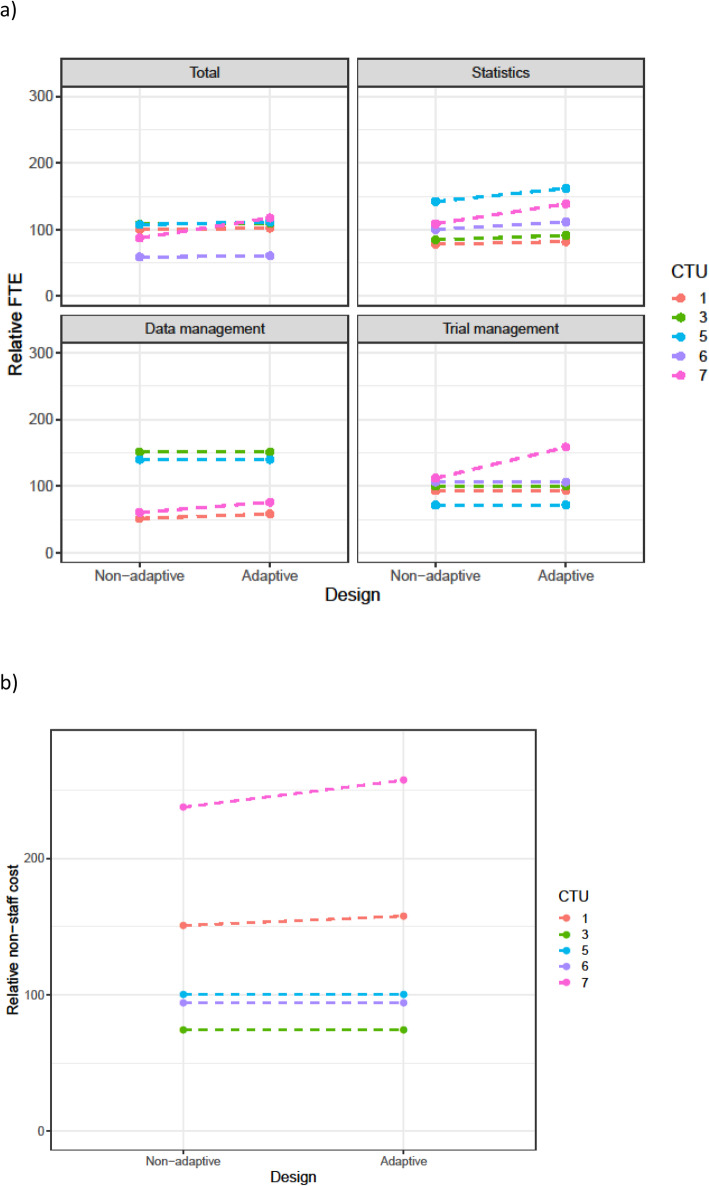
**a** FTE-years relative to non-adaptive median (set to 100) for scenario 4. **b** Total non-staff cost relative to non-adaptive median (set to 100) for scenario 4

**Fig. 5:**
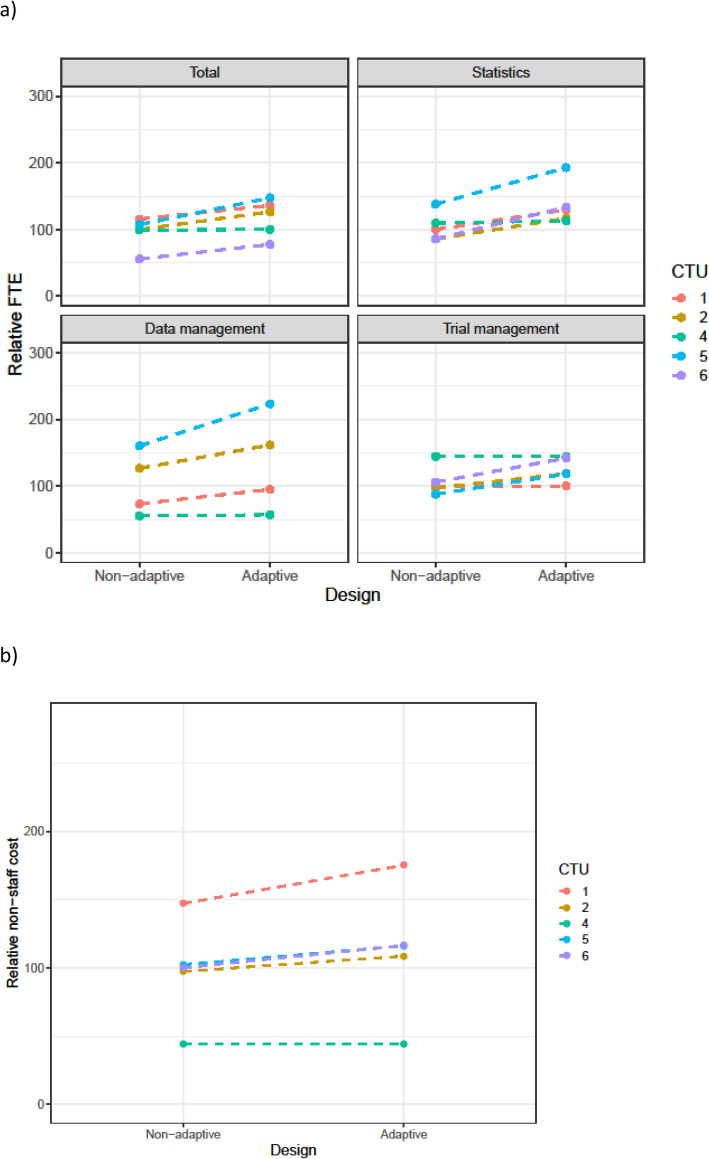
**a** FTE-years relative to non-adaptive median (set to 100) for scenario 5. **b** Total non-staff cost relative to non-adaptive median (set to 100) for scenario 5

Table 3 summarises the changes in staff resource that were requested for adaptive designs. This is split by scenario and by the main staff categories. Figures 1, 2, 3, 4, and 5 show the range in FTE-years for each scenario.

**Table 3:** Summary of the percentage increase in total FTE-years, FTE-years for statistics, data management and trial management, and non-staff costs between the adaptive over the non-adaptive version of each scenario

Scenario	% increase in total FTE-years, median (range)	% increase in statistics FTE-years, median (range)	% increase in data management FTE-years, median (range)	% increase in trial management FTE-years, median (range)	% increase in non-staff costs, median (range)
**1. Group-sequential design (*n*=6)**	3.9% (2.7%, 27.7%)	9.4% (2.3%, 34.2%)	4.8% (3.2%, 28.0%)	2.4% (2.0%, 29.9%)	0.8% (0.0%, 6.3%)
**2. Phase 2b dose-response (*n*=6)**	2.2% (0.7%, 17.5%)	13.4% (4.6%, 21.9%)	0.0% (0.0%, 6.6%)	0.0% (0.0%, 20.8%)	4.2% (0.0%, 8.2%)
**3. Phase 3 MAMS (*n*=5)**	3.0% (1.3%, 7.9%)	16.7% (4.7%, 26.0%)	9.6% (0.0%, 19.8%)	0.0% (0.0%, 14.2%)	0.8% (0.0%, 5.3%)
**4. Umbrella study (*n*=5)**	3.0% (1.0%, 34.2%)	11.1% (5.0%, 27.6%)	6.4% (0.0%, 25.0%)	0.0% (0.0%, 41.7%)	0.0% (0.0%, 8.3%)
**5. Sample size re-estimation (*n*=5)**	26.5% (0.8%, 38.9%)	36.8% (2.9%, 56.3%)	28.75% (2.9%, 39.3%)	22.2% (0.0%, 34.6%)	13.5% (0.8%, 19.0%)

*FTE* full-time equivalent, *MAMS* multi-arm multi-stage

Across all scenarios, resources increased for the adaptive design compared to the non-adaptive design. The median increase in staff resource was modest (2–4%) for all adaptive designs other than sample size re-estimation. However, this larger increase was driven by the increase in the length of the time needed for the adaptive trial. As discussed in the qualitative results section, CTU resource needs were calculated assuming the maximum project length would be achieved. Even for scenarios with low median increase, there was considerable variation across CTUs in this increase; this was explored further in the qualitative results section.

The resource needs for individual categories of staff demonstrate that across the scenarios, statistical resource generally increased the most, followed by data management and then trial management for the adaptive designs compared to their non-adaptive scenario. There was consensus across all CTUs, for all scenarios, that statistical resource should be increased for adaptive designs. The median statistics FTE-year increase for scenarios 1–4 was between 9 and 17%, with a larger increase for the sample size re-estimation design (scenario 5).

There was inconsistency on whether trial management resource should be increased for all scenarios, except the group sequential design where all CTUs increased the resource.

For data management, all CTUs increased resource for the group-sequential design, MAMS, and sample size re-estimation designs, but there were divergent responses for the phase 2b dose response and the umbrella study.

Changes in non-staff costs for each scenario are shown in panel (b) of Figs. 1, 2, 3, 4, and 5. All CTUs increased non-staff costs for the sample size re-estimation, but there was inconsistency for the other scenarios. Examples of non-staff costs that increased were randomisation and clinical data management system costs, patient and public involvement costs, and MHRA fees.

### Qualitative results

All seven CTUs that provided at least one costing also took part in an interview. Staff who did the costing exercise were interviewed individually or in pairs where they had worked together on the costing exercise. Eight interviews took place with 10 individuals (mean length 40 min; range 33–55 min). All interviews were conducted by KB, with two observed by JH. Two of the interviewees were known to the interviewer prior to the interview; the interviewer has a role in costing in a CTU and took part in the mock costing exercise.

#### Participant background

Interviewees were experienced in costing trials, with variation in their experience of costing adaptive designs. Individuals with more experience in adaptive designs tended to work on trials in oncology or rare diseases where these designs are more common.

[M]y main experiences around adaptive designs, and that’s becoming more common in cancer and I think it’s probably becoming more common in, in all areas now. 011

There was some difference in opinion about what an adaptive trial is and whether all scenarios were truly adaptive. This has been noted in other research [10].

So the scenario one I wouldn’t consider it adaptive…. Scenario two is a dose finding study, … it’s not an adaptive trial. Scenario three, I think I would say is adaptive. Scenario four, I don’t consider adaptive. And scenario five, I don’t consider adaptive. So there’s only one scenario I actually thought was an adaptive trial. 010

#### Costing exercise

Interviewees generally used a similar process for costing non-adaptive and adaptive designs, with some differences in who was involved in the costing.

The more complex a trial is, the more discussion you need to have with your colleagues to understand what it actually means for them. 014

Most CTUs had a template or model that they worked from and would use this to guide resource needs for all design types. A few CTUs had costing guidance in addition or instead of a template, and two interviewees said they did not use a template.

[W]e’ve got our costing model set-up, so almost, you know, when a trial comes in, its construction, whether it’s adaptive or not, it shouldn’t change the way we cost something. 011

In the adaptive versions of the trials, people considered the consequences of each adaptation during the costing process, whether they used a template or not.

I don’t use a proforma, it’s really just sitting down and working out what the different complexities are …. And that’s where sitting down with the Statisticians and the Pharmacists to work out what the impact of each of those adaptations... 010

I would say the approach would be really broadly the same. I think for a standard costing and for an adaptive costing you’re always thinking ok what, what might make this trial deviate from (yeah) the, the sort of norm, the expected and trying to think about any costs that might be incurred because of that. 015

In cases where the adaptive trial design would have a variable duration (e.g. group sequential design or sample size re-assessment), all interviewees costed for the ‘worst-case scenario’ to ensure the CTUs could deliver the whole trial. Most interviewees discussed the impact of not including enough resource in the costs, and that under-costing a study can adversely impact CTUs and their staff.

[O]ne of the huge benefits [of adaptive designs] is that you may be able to do things much more cost efficiently, because the study may stop early for whatever reason. And the difficulty is, from a costing perspective, you have to assume that at every step of an adaptation, the more costly and longer option is what will be selected. 010

Because of this, interviewees said they would present the highest cost to a funder but some added they might present a range of costs in the justification of costs in a finding application to show what the adaptive design would cost if it stopped at any of the pre-planned stages.

[I]f I was doing it for a funder then I might present both cases, the maximum (yeah) and what happens if it’s not the maximum, so sample size, re-estimation was an example where I provided two different adaptive costings depending on the minimum and maximum sample size required. 003

#### Resource needed

All interviewees increased statistics resource in the adaptive design scenarios, and the majority also increased data management (or related roles). Some increased trial management time, but not for every scenario. Increases in costs were mainly due to an increase in staff resource.

Actually, the methodologist role, sort of senior person we didn’t increase however, increased time for trial manager, senior trial manager, data manager most definitely and the trial administrator. 005

Sample size re-estimation increased the FTE-years and non-staff costs more on average than other scenarios, but this was due to the maximum project length increasing.

So, where you have a sample size re-estimation, [the funder doesn’t] necessarily want you to … go back for extra money. 010

The lack of experience with some designs and therefore the consequence of interim analyses may have led to an underestimate of the work involved.

[W]e don’t know if it did come to.. the interim analysis and we did have to drop an arm would that suddenly turn out to be .. much more work than we’d thought about for everybody else. 001

#### Statistics resource

Statistics resource (i.e. FTE-years) was always increased to undertake the interim analyses, and sometimes increased due to a higher workload during the development of the protocol and the statistical analysis plan.

So I think typically I would’ve put more time in for the design part of it… design went from two weeks to four weeks for the first scenario… Statistical analysis plan I put in two extra weeks for that to account for any interim analysis plan… and actually doing an interim analysis I put four weeks for that and probably put a slight extra bit for the analysis… the other tasks remained the same. 003

Some considered additional roles in relation to the need for an unblinded and a blinded statistician for the adaptive designs.

[A]re you going to need additional junior statisticians? Because are you going to have to have one that’s blinded and one unblinded? ... Because again, in most studies we’ll have a senior and junior statistician, but the moment you get to adaptive trials, you’re often thinking, ‘well actually, we need another person involved’ and then your costs are being pushed up. But they’re always so different. You can’t really standardise any of the process. You end up having to sit down one by one and figure it out. 010

Some interviewees increased the FTE-years of the statistician (and other staff) across the project, and some added additional resource at times of interim analysis and discussed how this might influence planning within the CTU.

We tend to run a model where even though we know there’s peaks and troughs within a trial, we do tend to flat line and try and make sure that workload is balanced across a range of projects at different stages… We tend to find [that] the easiest way to manage both staff and projects. 005

#### Trial management resource

CTUs that increased trial management time, usually by increasing the FTE across the trial, did so to cover increased complexity of the protocol and set-up tasks, the potential for increased protocol amendments, data cleaning, site monitoring, and other activity around interim analyses. There was variation between CTUs and scenarios on whether trial management resource should be increased.

But for, say, a trial manager, you don’t know if any one of those time points might result in a protocol amendment. The easier way to do it is just uplift it a little bit and smooth it, and as when you need that resource you manage it within your manpower within the unit. 014

When trial management resource was not increased, it was thought the trial manager could undertake the additional tasks required for the adaptive design within the FTE-years that they had been allocated for the non-adaptive design.

[D]epending on an adaptation, it may mean more frequent, or an adaptation may mean less frequent visits to sites. But if you've always costed at the maximum.... it shouldn't change your costing. 010

#### Data management/programmer resource

CTUs increased data management or related resource to undertake changes to the randomisation system or the database during the trial, or for increased activity around interim analyses, e.g. for data cleaning, database lock, and reporting.

[I]f you are dropping an arm for example [the randomisation system] needs to be adapted after that interim analysis. So you need … the time for the data manager to undertake that work. 005

CTUs that did not increase data management resource (or increased it by a small amount only) either thought the work would fit in with the resource already allocated, or tended to be those that were more experienced in making adaptations during their trials and therefore had systems set up to accommodate these changes, such as units experienced in oncology trials.

[I]f you’ve built the database in a way that reflects an adaptive design, you shouldn’t need to fiddle around with it too much whether it’s switched on or switched off. So a CTU that has got experience of this sort of design… the requirements shouldn’t be that different. 011

All CTUs increased data management resource for group-sequential design, MAMS, and sample size re-estimation but there was disagreement for the phase 2b dose response and the umbrella study.

I don’t think I’ve changed the duration on the second one [phase 2b]. So I don’t think the costs changed on that. 010

I think the umbrella or the MAMS design, where I just chose to uplift all of the job roles and that’s due to an element of uncertainty. So, some of the scenarios you can really focus in on what the tasks will be and what the consequences of the decision will be. Others, there’s so much uncertainty that I feel the only way to be able to deal with the variety of options is to uplift all FTEs. 014

#### Seniority or experience of staff for ADs

The majority of interviewees said they did not change seniority between adaptive and non-adaptive scenarios for any of the staff groups.

[W]e haven’t gone for a higher statistician… no we’ve kept the same grades that we would normally just with more time 001

And actually there is less to do sometimes within stats about seniority and more to do with experience in a particular design... So we tend to find … because it’s an adaptive design always need a senior. 005

In some cases, seniority was thought to be important in the complexity of the design or adaptation and may have varied between scenarios rather than between adaptive and non-adaptive versions.

In that case, I would put a higher-grade trial manager on it, because I would pre-empt that you need that level of expertise into the future. 014

A few interviewees increased the seniority of the statistician when costing the adaptive design compared to the non-adaptive design, due to the particular expertise or experience required.

[S]tatistician roles are kind of [less experienced grade], [more experienced grade] mostly and so I think I would be assigning more of the [more experienced grade] to the sort of key roles in the adaptive trials because of the impact on the design. 015

#### Other costs

There were other cost implications for adaptive designs discussed in the interviews. Drug manufacture was not included in the scenarios, but this would need to be considered for drug trials.

[T]he fact that if that now means a manufacturing step, you may have a two month, three month delay before you can actually implement that adaptation, because if that requires a decision about drug manufacturing to not be made until that interim, then they often won't have built in the timelines that are needed. 010

Other costs identified as important by at least one interviewee in costing adaptive designs were resources for additional monitoring visits and oversight meetings (including Patient and Public Involvement) due to extended timelines and interim analyses; resource needed for writing and publication fees for additional publications; and additional training and computer equipment. In some CTUs, randomisation systems and database costs incurred non-staff costs rather than staff costs, which increased for an adaptive design.

Some of the others, so, the umbrella study, MAMS design, I would potentially expect a longer set-up period. They are, in my experience, much harder to set up – the information you require during set-up is a lot higher. So, I would – the complexity of the study drives the set-up times. 014

All CTUs increased non-staff costs for the sample size re-estimation, but there was disagreement for the other scenarios.

[Y]ou wouldn’t have put any large differences for sort of data capture systems or randomisation systems cause yeah, they’re all in-house… 015

[F]or the non-staff costs, I think they changed a bit more, because part of the formula we’ve got in our costing is based on sample size, and you know, the length of time, so how many meetings and stuff you have, so that obviously changes. 011

#### Uncertainty and complexity

Those experienced in adaptive designs commented on the driver for costs relating to complexity and uncertainty rather than just being a trial that has pre-planned adaptations. This complexity or uncertainty also accounted for differences in increases between the non-adaptive and adaptive scenarios within a CTU.

What we do is very complex even if it’s not adaptive and so we think about the complexity and try to make sure that we’ve got the right level of resource in that initial costing. 012

#### CTU funding

CTUs had a range of different funding structures, leading to differences in the number of core (non-trial specific) funded staff, and other aspects of running a trial. Funding for CTUs included infrastructure from universities, public sector organisations (e.g. NIHR), and charities, which impacted on costs required for each individual trial.

[H]aving that kind of core infrastructure to lead on development activity is really important; we are so lucky to have it and I know that not all CTUs do. 012

[O]bviously our costs would be internally consistent for the adaptive and the non-adaptive but just in terms of the overall level of costings sort of reflect that unsupported environment in relative terms compared to, you know, having these other inputs. 015

## Discussion

Adaptive designs can provide considerable benefit to clinical trials; however, staff and non-staff resources required to support them have not been previously examined. This study, which used both quantitative and qualitative methods to explore the resource needs for a range of adaptive trial designs with academic CTUs in the UK, found that adaptive trials are generally thought to require more staff and non-staff resources than non-adaptive trials.

Resource needs in the mock costing exercise were generally included at the highest that would be needed, the so-called worst case scenario. This was to avoid, where possible, the requirement to return to the funder for additional funding, or the need for an academic institution to cover unfunded trial resource needs. This represents a potential limitation of most funders in the UK, where a single project length and cost is typically requested prior to the project being funded. Only one CTU described providing funders with multiple costs for different scenarios. This is illustrated by the sample size re-estimation scenario resulting in the highest increase in resources due to the potential for a substantial increase in project length if the adaptive design increases the sample size.

Those who had experience of working with adaptive trial designs in oncology and rare diseases were likely to be those with the most experience of the design, development, and delivery of adaptive trials. These units generally increased resources less for adaptive designs due to their increased familiarity with these designs that are more normalised in these disease areas.

Resources were consistently increased for statistical staff across every scenario. This reflected additional work required for more complex protocols and statistical analysis plans (perhaps including a simulation study to inform the design of the trial), alongside the time needed to undertake the interim analyses. There was wider variability in the need for additional staff resource beyond statistics. The data and trial management time was not increased for some scenarios; however, the wide ranges indicate that this is not consistent between units, and may be a reflection of the ‘worst case scenario’ costing and the complexity of the non-adaptive design in each scenario.

The complexity of the non-adaptive design in each scenario may also explain the variability between scenarios. As an example, the non-adaptive dose response (scenario 2) and umbrella design (scenario 4) were seen as complex even without an adaptive design. The non-adaptive umbrella design is effectively two trials run under the same protocol, so in itself provides advantages over running two separate trials. It should also be noted that not all scenarios were costed by every CTU, which may additionally explain some differences in variability between scenarios.

The approach to costings differed between, and in some cases within, units depending on the staff involved in costing. The two main approaches involved using individual tasks to generate the associated FTE, and experience based. Most trials units chose to flatten the percentage FTE to give a single figure across the trial. Differences in contexts and processes across CTUs mean it is unlikely the variability in additional resources required for adaptive designs would ever completely disappear.

The requirements for extra non-staff costs related mostly to additional monitoring visits and database and randomisation system costs. There is great variation across CTUs in the systems used; those that had systems that had been developed in-house did not require additional costs to implement the required adaptive elements to the trials (although they may still have needed additional staff resource).

It is clear that adaptive designs are often more complex to design and deliver than their non-adaptive counterparts. The uncertainty about whether trials will, for example, require an increased sample size, increased time, or other pre-planned changes may raise their perceived financial risk level for CTUs that are less experienced in their design and delivery.

One notable type of adaptive design that was not thoroughly considered is Response Adaptive Randomisation (RAR), in which allocation probabilities to treatment arms are changed according to patient outcomes observed. One area that RAR has been used in is multi-arm and umbrella trials, such as I-SPY2 [20]. Scenario 2 involved changing the randomisation allocation to different doses at an interim analysis and therefore has some similarities to RAR. However, designs like I-SPY2 assume continual change in allocation after each patient’s outcome is observed, whereas scenario 2’s adaptive design included only one change. Interestingly, scenario 2 had the highest change in median non-staff costs (except for sample size re-assessment), due to the change in randomisation required. Having continual change in randomisation probabilities during the trial would likely increase these costs further.

We would highlight that the median increase in cost compares favourably against the increase in efficiency that some adaptive designs can provide. As an example, even a two-stage group-sequential design can reduce the average sample size used by up to 35% [21] with more interims reducing this further (see e.g. [22]). This maximum gain is impacted by the choice of design, the true treatment effect, and the delay in how long it takes to assess the primary outcome [23, 24]. Other adaptive designs can increase the average power of the trial, improve patient benefit, or ensure the trial is robust to uncertainties as the design stage. It is difficult to quantify these advantages provided to directly compare to the increased cost, and more research is required on this.

### Limitations

Although this research has provided useful information, there are several limitations. The resource exercise focussed on CTU resources and did not explore other research costs such as resources for drug/intervention supply, or other methodological groups that may be involved in a trial, for example health economists or researchers using qualitative methods. The results are derived from seven CTUs in the UK which ranged from moderate size to amongst the largest in the UK. Five of the seven CTUs had researchers involved in the CAT grant application, although in only one case was a grant co-applicant the staff member who did any costings. Anecdotally, CTUs that had not previously run an adaptive trial were less likely to take part from the 53 invited to participate. Some CTU staff who took part were open that they had less experience in adaptive designs and may not fully understand the implications on resources required.

Theoretical scenarios were provided without the wider context of the full application, and without opportunity for refinement of resource needs based on discussion and feedback inherent to trial development. There was recognition in the interviews that final resource needs would involve a process of discussion and refinement. Without repeating the exercise or engaging in re-running the exercise including a discussion element, it will not be known what impact this may have had on the resources included. The scenarios covered trials of differing durations, making it difficult to draw direct comparisons between scenarios. The costing spreadsheet provided (Additional file 2) may have limited the scope of providing resource estimates, although was not reported as a barrier in the interviews.

The CTUs included in the project all operate autonomously, as such the operational structures differ, including with differing job roles and titles. Where possible, job roles were combined for the analysis, to enable comparison and protect the anonymity of units and individuals contributing to the resourcing exercise. This may lead to subtlety in some roles being lost (e.g. a statistical programmer role could have some data management duties as well as statistical).

### Further research and guidance

This is the first piece of research to systematically gather and analyse prospective data on resourcing clinical trials utilising an adaptive design. Whilst we have seen large variance in our sample, we have also identified a consistent and clear need for (typically modest) additional resource, most notably in statistical support. We would recommend that a similar exercise is conducted again in 3–5 years as the field continues to evolve, to ensure that trialists, investigators, and funders understand the resource needs of adaptive trials, and to refine the guidance as the efficiencies of the designs become further embedded in a wider number of therapeutic areas and trials units. This exercise could include methodological disciplines not included in this exercise, such as health economists and qualitative researchers (who are not typically based in CTUs). The exercise could also benefit from improved reporting of adaptive trials that would result from the adaptive designs CONSORT extension [4].

In conjunction with this paper summarising the research, we are developing a guidance paper for researchers who wish to resource an adaptive trial. This will also include a template costing tool representing all the tasks required for an adaptive trial. It will also contain recommendations for funders of trials that may allow more transparent, informative costings for adaptive trials that would ensure adaptive designs can continue to increase in use.

As mentioned earlier, further research to better quantify the benefits of adaptive designs and allow equating it to the increased costs identified here would be useful. There is also a need for further development of trials methods that could be used to reduce the additional cost, for example to do interim analyses more efficiently. Such methods could be implemented and investigated in trials using a study within a trial (SWAT) to ensure any positive and negative impacts of the method are measured.

## Conclusions

Adaptive designs provide convincing advantages in many situations. Findings from this research indicate that adaptive trials may require more staff and non-staff resources than non-adaptive trials, at least in the ‘worst case scenario’. Further research to examine how to weigh-up the advantages against the additional resource would help ensure that adaptive designs are used when they are likely to provide benefit. Additional research that could help reduce the gap in resources required between non-adaptive and adaptive trials would help increase the number of situations when the latter are cost-effective.

## Supplementary Information

**Additional file 1.** Scenarios and guidance pre-revision.

**Additional file 2.** Resource template pre-revision.

## Data Availability

The datasets generated and/or analysed during the current study are not publicly available due to anonymity reasons; requests for data can be submitted to the corresponding author and will be considered subject to not compromising anonymity.

## Abbreviations

CAT: Costing Adaptive Trials
CTU: Clinical Trials Unit
FEC: Full economic costing
FTE: Full time equivalent
MAMS: Multi-arm multi-stage
MHRA: Medicines and Healthcare products Regulatory Agency
NAD: Nicotinic acid derivative
OHSS: Ovarian hyper-stimulation syndrome
PICO: Participants, Intervention(s), Comparator, Outcomes
PPI: Patient and public involvement
UKCRC: UK Clinical Research Collaboration

## References

[1] DiMasi JA, Grabowski HG, Hansen RW (2016). Innovation in the pharmaceutical industry: new estimates of R&D costs. J Health Econ..

[2] Bentley C, Cressman S, van der Hoek K, Arts K, Dancey J, Peacock S (2019). Conducting clinical trials—costs, impacts, and the value of clinical trials networks: a scoping review. Clin Trials..

[3] Pallmann P, Bedding AW, Choodari-Oskooei B, Dimairo M, Flight L, Hampson LV, Holmes J, Mander AP, Odondi L’, Sydes MR, Villar SS, Wason JMS, Weir CJ, Wheeler GM, Yap C, Jaki T (2018). Adaptive designs in clinical trials: why use them, and how to run and report them. BMC Med..

[4] Dimairo M, Pallmann P, Wason J, Todd S, Jaki T, Julious SA, Mander AP, Weir CJ, Koenig F, Walton MK, Nicholl JP, Coates E, Biggs K, Hamasaki T, Proschan MA, Scott JA, Ando Y, Hind D, Altman DG (2020). The Adaptive designs CONSORT Extension (ACE) statement: a checklist with explanation and elaboration guideline for reporting randomised trials that use an adaptive design. BMJ..

[5] Burnett T, Mozgunov P, Pallmann P, Villar SS, Wheeler GM, Jaki T (2020). Adding flexibility to clinical trial designs: an example-based guide to the practical use of adaptive designs. BMC Med..

[6] Hatfield I, Allison A, Flight L, Julious SA, Dimairo M. Adaptive designs undertaken in clinical research: a review of registered clinical trials. Trials. 2016;17(150):1–13. 10.1186/s13063-016-1273-9 PMC4799596 26993469

[7] Sato A, Shimura M, Gosho M (2018). Practical characteristics of adaptive design in phase 2 and 3 clinical trials. J Clin Pharm Ther..

[8] Bothwell LE, Avorn J, Khan NF, Kesselheim AS (2018). Adaptive design clinical trials: a review of the literature and ClinicalTrials.gov. BMJ Open.

[9] Jaki T (2013). Uptake of novel statistical methods for early-phase clinical studies in the UK public sector. Clin Trials..

[10] Dimairo M, Boote J, Julious SA, Nicholl JP, Todd S (2015). Missing steps in a staircase: a qualitative study of the perspectives of key stakeholders on the use of adaptive designs in confirmatory trials. Trials..

[11] Madani Kia T, Marshall JC, Murthy S (2020). Stakeholder perspectives on adaptive clinical trials: A scoping review. Trials..

[12] Stallard N, Hampson L, Benda N, Brannath W, Burnett T, Friede T, Kimani PK, Koenig F, Krisam J, Mozgunov P, Posch M, Wason J, Wassmer G, Whitehead J, Williamson SF, Zohar S, Jaki T (2020). Efficient adaptive designs for clinical trials of interventions for COVID-19. Stat Biopharm Res..

[13] Speich B, von Niederhäusern B, Schur N, Hemkens LG, Fürst T, Bhatnagar N, Alturki R, Agarwal A, Kasenda B, Pauli-Magnus C, Schwenkglenks M, Briel M, MAking Randomized Trials Affordable (MARTA) Group (2018). Systematic review on costs and resource use of randomized clinical trials shows a lack of transparent and comprehensive data. J Clin Epidemiol..

[14] Hind D, Reeves BC, Bathers S, Bray C, Corkhill A, Hayward C, Harper L, Napp V, Norrie J, Speed C, Tremain L, Keat N, Bradburn M (2017). Comparative costs and activity from a sample of UK clinical trials units. Trials..

[15] Speich B, Schur N, Gryaznov D, von Niederhäusern B, Hemkens LG, Schandelmaier S, Amstutz A, Kasenda B, Pauli-Magnus C, Ojeda-Ruiz E, Tomonaga Y, McCord K, Nordmann A, von Elm E, Briel M, Schwenkglenks M, a collaboration of the MARTA (MAking Randomized Trials Affordable) and ASPIRE (Adherence to Standard Protocol Items: REcommendations for interventional trials) Study Groups (2019). Resource use, costs, and approval times for planning and preparing a randomized clinical trial before and after the implementation of the new Swiss human research legislation. PLOS ONE..

[16] Nevens H, Harrison J, Vrijens F, Verleye L, Stocquart N, Marynen E, Hulstaert F (2019). Budgeting of non-commercial clinical trials: development of a budget tool by a public funding agency. Trials..

[17] Wickham H (2009). ggplot2: Elegant Graphics for Data Analysis.

[18] R: The R Project for Statistical Computing. [Accessed 26 May 2021]. Available from: https://www.r-project.org/

[19] Jane R, Liz S. Qualitative Data Analysis for Applied Policy Research. In: The Qualitative Researcher’s Companion. Thousand Oaks: SAGE Publications, Inc. 2002. p 305–29. Available from: http://methods.sagepub.com/book/the-qualitative-researchers-companion/n12.xml.

[20] Barker AD, Sigman CC, Kelloff GJ, Hylton NM, Berry DA, Esserman LJ (2009). I-SPY 2: an adaptive breast cancer trial design in the setting of neoadjuvant chemotherapy. Clin Pharmacol Ther..

[21] Wason JMS, Mander AP (2012). Minimizing the maximum expected sample size in two-stage phase II clinical trials with continuous outcomes. J Biopharm Stat..

[22] Wason JMS, Mander AP, Thompson SG (2012). Optimal multi-stage designs for randomised clinical trials with continuous outcomes. Stat Med..

[23] Hampson LV, Jennison C (2013). Group sequential tests for delayed responses. J R Stat Soc B..

[24] Wason JMS, Brocklehurst P, Yap C (2019). When to keep it simple – adaptive designs are not always useful. BMC Med..

