# Supplementary material for: Costs and staffing resource requirements for adaptive clinical trials: quantitative and qualitative results from the Costing Adaptive Trials project
Source: BMC Med. 2021 Oct 26;19:251. doi: 10.1186/s12916-021-02124-z (PMC8545558; doi:10.1186/s12916-021-02124-z)
Supplement: Supplementary file 1 — Additional file 1. Scenarios and guidance pre-revision. [file 12916_2021_2124_MOESM1_ESM.docx]

**Scenario 1 - Two-arm randomised controlled trial assessing addition of biomarker-testing to an existing early warning score in the management of patients with suspected sepsis in the emergency department**

**Brief synopsis**: Sepsis is a medical emergency; early use of broad-spectrum antibiotics is an important part of management. Currently some patients are inappropriately diagnosed as having sepsis, leading to inappropriate antibiotic use and consequent adverse events. Some patients may have sepsis but are not correctly diagnosed, resulting in under-treatment. This trial will test whether introducing monitoring of a biomarker of sepsis will reduce inappropriate antibiotic prescribing in patients with suspected sepsis.

**Participants and sites**: Patients >=18 years, presenting to Accident and Emergency (A&E) with suspected sepsis. Suspected sepsis will be identified at A&E and after initial administration of a routinely used early warning score (EWS), patients will be assessed for inclusion/exclusion criteria; those who meet them will be recruited and randomised using a deferred consent model.

A total of 8 NHS sites across all regions of England and Wales will be recruited from.

**Intervention**: Patients who are randomised to the biomarker group will (within an hour) undergo assessment using a point-of-care test as well as routine EWS assessment. Using a pre-specified risk algorithm combining the biomarker and EWS, patients will be treated as ‘high risk’, ‘moderate risk’ or ‘low risk’.

**Comparator**: Based on pre-specified criteria in routine use, patients will be classed as ‘high risk’, ‘moderate risk’ or ‘low risk’ based on the EWS alone.

High risk patients (from both arms) will be referred to IV antibiotics within an hour; moderate risk patients will be reviewed within 3 hours with IV antibiotics considered; low risk patients will be managed according to clinical judgement.

**Outcomes**: patients will be followed up for at least 90 days.

Primary outcome: 30-day all-cause mortality.

Secondary outcomes:

- initiation of IV antibiotics within 3 hours of randomisation;

- time until initiation of IV antibiotics

- Number of days on IV antibiotics

- ICU admission

- Adverse antibiotic outcomes

- Readmission to hospital within 90 days of randomisation

- Mortality within 90 days of randomisation

- EQ-5D/5L at 90 days (to be analysed by health economist co-applicant)

**Research team**

The co-applicants currently include a chief-investigator, the 7 site PIs (3 of whom are also clinical academics), a patient representative and a health economist. The team should also include a senior statistician and suitable senior clinical trial methodologist co-applicant (which should be included in the resourcing).

**Trial committees**

Safety data and outcome data are to be reviewed at six-monthly Independent Data Monitoring Committee (IDMC) meetings. There will be a six-monthly Trial Steering Committee (TSC) with all co-investigators invited and with three independent members. A trial management group (TMG) will take place regularly. There will be a PPI representative on the TSC and TMG.

**Non-adaptive design**

A total of 6500 patients will be recruited, powered to show non-inferiority of the biomarker arm to the control arm on 30-day mortality. This has been inflated to allow for 5% dropout rate. A total of 26 months recruitment will be assumed, with patients to be followed-up for at least 90 days.

Patients will be randomised in a 1:1 ratio, stratified by baseline EWS and centre. Stratified permuted block randomisation with random block sizes will be used. A 24 hour web randomisation system controlled centrally by the CTU will be used.

Data will be analysed through suitable regression models that account for baseline stratification variables. As well as a primary intention to treat analysis, a per-protocol analysis will exclude patients in whom the EWS and/or biomarker score were not used in treatment decisions.

**Adaptive design**

A maximum total of 6700 patients will be recruited over 27 months. After the first half have had their 30-day mortality outcome measured, an interim analysis will be conducted. The trial will be stopped early for likely lack of non-inferiority if the one-sided p-value for non-inferiority from the first half of patients is greater than 0.5. Detailed results from the interim analysis would only be seen by the IDMC and the trial statistician. The IDMC meeting to discuss the interim analysis results will replace one of the six-monthly IDMC meetings. The trial will not be paused during the interim analysis and decision-making process. The protocol will be amended as a result of the interim analysis.

Otherwise the design is as in the ‘non-adaptive’ section.

**Trial Timelines**

Non-adaptive: Nine months for trial set-up and opening of initial three centres; 26 months recruitment; 3 months follow-up; six months data cleaning, analysis, reporting.

Adaptive: Nine months for trial set-up and opening of initial three centres; maximum of 27 months recruitment (depending on results of interim analysis); 3 months follow-up; six months data cleaning, analysis, reporting.

**Scenario 2 - A randomised dose-finding study of JAK1 inhibitor for patients with active rheumatoid arthritis**

**Brief background**: Methotrexate has been successful in improving outcomes in many rheumatoid arthritis (RA) patients. However, a sizeable proportion of patients fail to respond adequately and there is a need for new treatments in this population. Janus kinase (JAK) inhibitors show promising early phase trial results as an efficacious second-line therapy for RA. This trial will test four doses of JAK1 inhibitor against placebo in order to identify the optimal regimen for a subsequent phase III trial.

**Participants and sites**: Adults (>=18 years) who have been diagnosed with RA for at least six months and have shown inadequate response to methotrexate (and who agree to be washed out from methotrexate for at least a month). Patients are allowed to be on non-steroidal anti-inflammatory drugs and oral glucocorticoids if their dosage was stable for the previous month. Patients are excluded if they are currently receiving any other disease-modifying antirheumatic drug (DMARD).

Patients will be recruited across 12 sites in England, Wales and Scotland. It is estimated that the recruitment will take 2 years.

**Intervention**: The experimental arms are 25, 50, 100 or 200 mg JAK1 inhibitor (one tablet, twice daily). Treatment is given until week 24.

**Comparator**: Patients randomised to the control arm will be treated with placebo (matched tablet given twice daily) for 24 weeks.

**Outcomes**

Patients will be followed up every 4 weeks until week 24 for efficacy, disease activity and safety endpoints.

Primary outcome: the primary outcome is responder status at 12 weeks as classified by the ACR20, which classifies patients by tender and swollen joint counts, and patient’s global assessment of disease activity.

Secondary outcomes (all at each time point):

- ACR50 and ACR70 response

- Disease Activity Scale (DAS28)

- Clinical Disease Activity Index (CDAI).

- Health-related quality of life

- Adverse events.

**Research team**

The co-applicants currently include a chief-investigator (clinical academic), 4 other clinical academics and a pharmacist. The team should also include a senior statistician and suitable senior clinical trial methodologist co-applicant (which should be included in the resourcing)

**Trial committees**

Safety data and outcome data is to be reviewed at six-monthly Independent Data Monitoring Committee (IDMC) meetings that should consist of three independent members. There will be a six-monthly Trial Steering Committee (TSC) with all co-investigators invited and with three independent members. A trial management group will take place regularly. There will be a PPI representative on the TSC and TMG.

**Non-adaptive design**

A total of 250 patients will be recruited and randomised 1:1:1:1:1 between the placebo, 25, 50, 100 or 200 mg arms. The randomisation will be stratified by prior use of a biological DMARD. Block randomisation with random block sizes will be used. A 24 hour web randomisation system controlled centrally by the CTU will be used.

This sample size was chosen to provide sufficient information to estimate the ED50 (effective dose giving 50% of maximum efficacy) with a target level of precision. It will also provide sufficient power to test each dose arm against placebo for superiority. It is expected based on similar trials that around 5% of patients will dropout by 12 weeks.

For the dose-response analysis, data will be analysed through fitting a suitable dose-response model such as an Emax model. For superiority testing, suitable regression models that account for baseline measurements will be used. As well as a primary intention to treat analysis, a per-protocol analysis will exclude patients in whom compliance with treatment was below 75% as assessed with pill counts.

**Adaptive design**

After 125 patients have their 12 week efficacy assessment, an interim analysis will commence (during which recruitment will continue). The interim analysis will fit a three parameter Emax model to the outcome data assessed so far, and use this to find an optimal design for the second stage of the study to maximise the dose-response information found. Placebo will be counted as a dose of 0 in this analysis. This analysis will likely lead to a change in the proportion of patients randomised to each dose (but existing dose level will not be changed). Allocation to placebo will be set to a minimum of 20% in the second stage.

The detailed results of the interim analysis would be reviewed by the IDMC. Only the trial statistician and the IDMC will see unblinded data. The IDMC meeting to discuss the interim analysis results will replace one of the six-monthly IDMC meetings. The trial will not be paused during the interim analysis and decision making process. If the allocation is changed as a result of the interim analysis, the protocol will be amended.

Otherwise the design will be as in the non-adaptive design section.

**Trial Timelines**

Non-adaptive: Nine months for trial set-up and opening of initial four centres; 24 months recruitment; 3 months follow-up; six months data cleaning, analysis, reporting.

Adaptive: as above.

**Scenario 3 - A multi-arm open-label phase 3 trial comparing regimens for treating intermediate and high-risk oropharyngeal cancer**

**Brief synopsis**: Patients with intermediate or high-risk oropharyngeal cancer have poor prognosis, with three year survival rates of 70% and 45% respectively. There is a need for new treatment strategies, which may include different regimens of chemotherapy and new drugs. This trial will test three promising experimental interventions against current gold-standard chemotherapy to determine if any provide longer term overall survival advantages.

**Participants and sites**: Patients(18-70 years old) with intermediate or high-risk oropharyngeal cancer, as classified by Human Papillomavirus (HPV) status and smoking history with minimum life expectancy of 4 months and Eastern Cooperative Oncology Group (ECOG) status 0 or 1.

Patients will be recruited from a total of 22 NHS sites across England, Scotland and Wales.

**Intervention arms**

1. Induction (higher intensity) chemotherapy for 3 cycles at 4-weekly intervals followed by standard chemotherapy and radiotherapy as per control arm.
2. Tumour resection followed by standard chemotherapy and radiotherapy as per control arm.
3. Induction immunotherapy followed by standard chemotherapy and radiotherapy as per control arm.

**Comparator**

The control arm will be the international gold-standard chemoradiotherapy regimen, which is three three-weekly cycles of cisplatin and concomitant radiotherapy for 7 weeks.

**Outcomes**

Patients will be followed-up monthly for at least 1 year followed by annual follow-up for up to 5 years, or until death.

Primary outcome: the primary outcome is overall survival (OS).

Secondary outcomes

- Event-free survival (event is death, distant metastasis, persistent disease three months after completion of treatment, clinical or radiological relapse/recurrence).

- Total number of grade 3+ toxicity events up to 1 year post randomisation.

- Quality of life measurement at 1 year post randomisation

- Surgical complication rates

- Cost effectiveness as assessed with EQ-5D.

**Research team**

The co-applicants include a chief-investigator, five clinical academics, a patient representative and a qualitative researcher focused on improving recruitment rates. The team should also include a senior statistician and suitable senior clinical trial methodologist co-applicant (which should be included in the resourcing).

**Trial committees**

Safety data and outcome data are to be reviewed at six-monthly Independent Data Monitoring Committee (IDMC) meetings. There will be a six-monthly Trial Steering Committee (TSC) with all co-investigators invited and three independent members. A trial management group (TMG) will take place regularly. There will be a PPI representative on the TSC and TMG.

**Non-adaptive design**

This trial will have a five year recruitment period and each patient will be followed up for at least one year. The final analysis will take place once all patients have reached one year follow-up, at which point it is anticipated there would be 600 patients recruited. Randomisation is equal between arms (1:1:1:1), stratified by HPV infection history and smoking history (never, ex-smoker, current smoker).

A yearly IDMC meeting will take place to review safety data.

Each arm will be analysed against control using suitable regression models that account for baseline stratification variables. Cox regression will be used for the primary outcome and secondary time-to-event outcomes. The primary analysis is intention to treat, with a secondary per-protocol analysis excluding patients who did not receive the allocated treatment.

**Adaptive design**

A multi-arm multi-stage design will be used with two interim analyses (scheduled 2 years and 4 years into the recruitment period). Event-free-survival will be compared between each experimental arm and control and the resulting test statistics used to allow early stopping of less promising arms. At the first interim, any experimental arm with a hazard ratio of >1 compared to control will be dropped; at the second interim, any experimental arm with a hazard ratio of >0.9 will be dropped. When an arm is dropped patients are still followed up.

If experimental arms are dropped, the randomisation will be equal between control and remaining experimental arms. If all experimental arms are dropped at an interim analysis, the trial will finish early although all current patients will be followed up for at least a year. It is anticipated that this is unlikely. The trial will continue to the planned 600 patient enrolment unless all arms are stopped early.

Results of interim analyses will be ratified by the IDMEC, who will make recommendations to the TSC about which arms should be stopped. Only the trial statistician and IDMEC will see unblinded data. The IDMC meetings to discuss the interim analyses results will be scheduled to take place at the same time as the planned annual IDMC meetings. The trial will not be paused during the interim analysis and decision making process. If arms are dropped, the protocol will be amended as a result of the interim analysis.

Otherwise the design is as in the ‘non-adaptive’ section.

**Trial Timelines**

Non-adaptive: Nine months for trial set-up and opening of initial six centres; 60 months recruitment; 12 months minimum follow-up; six months data cleaning, analysis, reporting.

Adaptive: As above, unless all experimental arms stop at first interim or second interim analysis.

**Scenario 4 - A randomised controlled trial assessing clinical and cost-effectiveness of earlier treatment of ovarian hyper-stimulation syndrome**

**Brief synopsis**: Ovarian Hyper-Stimulation Syndrome (OHSS) is a serious side-effect of reproductive treatments that involve stimulating the ovaries. OHSS is classified into early and late that each have separate causes. Current practice is to treat OHSS only once it progresses to a severe OHSS; this generally leads to the patient requiring intensive inpatient treatment. Two treatments have shown some potential promise in small uncontrolled studies at preventing the need for hospitalisation: 1) draining of the ascites; 2) currently licensed GnRH antagonists. This trial will be an ‘umbrella study’ and consist of two separate sub-trials: 1) a three-arm trial comparing draining and GnRH antagonist to usual care in early OHSS; 2) a two-arm study comparing draining to usual care in late OHSS.

**Participants and sites**: Women with moderate or severe early or late OHSS will be recruited from 18 NHS and private fertility clinics in the UK. Only women who have not yet had embryo transfer or who have a negative pregnancy test will be included.

**Intervention arms**

For early OHSS:

1. Draining of the ascites with route of entry either vaginally or abdominally in an outpatient setting.
2. GnRH antagonists, administered once daily for four days using subcutaneous injection. These drugs are routinely available in IVF clinics and are licensed for use.

For late OHSS:

1. Draining of the ascites with route of entry either vaginally or abdominally in an outpatient setting.

**Comparator**

The standard of care arm will involve following standard management of OHSS, which includes fluid management, thromboprophylaxis and analgesia.

**Outcomes**

Patients will be followed-up for 28 days (and subsequently for birth outcomes).

Primary outcome: the primary outcome is any OHSS related hospitalisation within 28 days.

Secondary outcomes:

- Time to resolution of OHSS
- Patient satisfaction using CSQ-8.
- Any adverse event until 28 days.
- Time until progression in OHSS (from moderate to severe or severe to critical).
- Quality of life using EQ-5D-5L.

**Research team**

The co-applicants include a chief-investigator, four clinical academics, a patient representative and a qualitative researcher. The team should also include a senior statistician and suitable senior clinical trial methodologist co-applicant (which should be included in the resourcing)

**Trial committees**

Safety data and outcome data are to be reviewed at six-monthly Independent Data Monitoring Committee (IDMC) meetings. There will be a six-monthly Trial Steering Committee (TSC) with all co-investigators invited and with three independent members. A trial management group (TMG) will take place regularly. There will be a PPI representative on the TSC and TMG.

**Non-adaptive design**

The early OHSS sub-trial will randomise 240 patients equally between the three arms. It is anticipated that the recruitment period will be 2 years in total, with all patients followed up for 28 days.

Randomisation will be stratified block randomisation with stratification by severity of OHSS (moderate or severe).

The late OHSS sub-trial will randomise 80 patients equally between the two arms. This is anticipated to also take 2 years as the condition is rarer. Follow-up will be similar to above.

The two sub-trials are run in parallel and will be analysed separately. Each experimental arm will be compared to control using suitable regression models that account for the baseline stratification variable. Logistic regression will be used for the primary outcome and secondary binary endpoints. Cox regression will be used for time-to-event outcomes. The primary analysis is intention to treat, with a secondary per-protocol analysis.

**Adaptive design**

In the early OHSS sub-trial, an interim analysis will allow recruitment to a non-promising arm to be stopped early. The interim analysis will take place once 90 patients have 28 day outcome data measured (expected to be one year into the recruitment period). Any experimental arm that has a one-sided p-value (for 28 day OHSS-related hospitalisation) above 0.4 will be deemed to be non-promising and dropped from the trial. If both experimental arms are non-promising, the trial will finish early; if one is non-promising the target sample size will be reduced to 200.

In the late OHSS sub-trial, an interim analysis will test the experimental vs control arm. If the one-sided p-value is above 0.5, this sub-trial will finish early for futility.

The IDMC meeting to discuss the interim analysis results will replace one of the six-monthly IDMC meetings. The trial will not be paused during the interim analysis and decision making process.

The protocol will be amended as a result of the interim analysis if arms are dropped from either sub-trial.

Otherwise the design is as in the ‘non-adaptive’ section.

**Trial Timelines**

Non-adaptive: Nine months for trial set-up and opening of initial six sites; 24 months recruitment; one-month follow-up; six months data cleaning, analysis, reporting.

Adaptive: As above, unless trial terminates or sample size target reduced at interim analysis.

**Scenario 5 - Randomised double-blinded placebo-controlled trial of the efficacy of nicotinic acid derivative (NAD) for treatment of fatigue in mitochondrial disease**

**Brief synopsis**: Adult patients with mitochondrial disease (MD) often experience fatigue, muscle weakness and reduced exercise tolerance. There are currently no interventions that have the realistic prospect of cure. A generically available drug derived from nicotinic acid (NAD) has shown great promise in pre-clinical studies for targeting the biological pathway causing this symptom. This will be a trial aimed at testing early efficacy of the drug in MD patients.

**Participants and sites**: Adults (>=18 years) with MD who are enrolled in the MRC Mitochondrial Cohort, which contains >1000 UK registered patients. Patients will be treated in three centres across England.

**Intervention**: Patients who are randomised to the experimental arm will be treated with NAD tablet twice daily for 8 weeks as well as standard of care.

**Comparator**: Patients randomised to the control arm will be treated with standard of care and 8 weeks of matched placebo tablet twice daily.

**Outcomes**: patients will be followed up for the 8-week treatment period.

Primary outcome: the primary outcome is change from baseline to 8 weeks in ATP content in muscle biopsy specimens.

Secondary outcomes (all measured at baseline and at 8 weeks):

- perceived fatigue (fatigue severity scale)

- cardiopulmonary fitness (VO2 peak)

- disease burden (Newcastle Mitochondrial Disease Adult Scale)

- 10 metre timed walk

- formal gait analysis (Gaitrite)

- daily physical activity measured by accelerometers.

**Research team**

The co-applicants currently include a chief-investigator (clinical academic), the 2 other site PIs (both clinical academics), a pharmacist, an international academic expert in the clinical area. The team should also include a senior statistician and suitable senior clinical trial methodologist co-applicant (which should be included in the resourcing).

**Trial committees**

Safety data and outcome data are to be reviewed at six-monthly Independent Data Monitoring Committee (IDMC) meetings. There will be a six-monthly Trial Steering Committee with all co-investigators invited and three independent members. A trial management group will take place regularly. There will be a PPI representative on the TSC and TMG.

**Non-adaptive design**

A total of 100 patients will be recruited and randomised 1:1 between the NAD and placebo arms. This is powered to show superiority of the NAD arm on the primary outcome. This has been inflated to allow for 10% dropout rate. Once the three sites are open, it is expected that they will each recruit 1 patient per month on average.

Patients will be randomised in a 1:1 ratio stratified by centre. Block randomisation with random block sizes will be used. A 24 hour web randomisation system controlled centrally by the CTU will be used.

Data will be analysed through suitable regression models that account for baseline measurements. As well as a primary intention to treat analysis, a per-protocol analysis will exclude patients in whom compliance with treatment was below 50% as assessed with pill counts.

**Adaptive design**

Since there is little prior information about the standard deviation of the outcome, a sample-size re-estimation design is to be used.

After 80 patients have their 8-week primary outcome measured, an interim analysis will be conducted. A blinded analysis will estimate the pooled standard deviation and assess what sample size would be required for 80% conditional power. If this is between 80 and 150, recruitment will continue until that number are randomised. If greater than 150, the trial will be stopped early for likely lack of power. In that case no further patients will be recruited, although all patients recruited to that point the decision was made will be treated and the trial will be analysed with the reduced number. The IDMC meeting to discuss the interim analysis results will replace one of the six-monthly IDMC meetings. The trial will not be paused during the interim analysis and decision making process. The protocol will be amended as a result of the interim analysis.

The data will be analysed as described in the non-adaptive section regardless of the final sample size.

The new sample size will be communicated to all investigators. The protocol will be amended as a result of the interim analysis if the sample size changes.

Otherwise the design is as in the ‘non-adaptive’ section.

**Trial Timelines**

Non-adaptive: Nine months for trial set-up and opening of all three centres; 36 months recruitment; two months follow-up; six months data cleaning, analysis, reporting.

Adaptive: Nine months for trial set-up and opening of all three centres; minimum of 30, maximum of 54 months recruitment (depending on results of interim analysis); two months follow-up; six months data cleaning, analysis, reporting.

**Costing Adaptive Trials - Instructions and guidance**

Thank you very much for agreeing to contribute to this project.

The main objectives of the research are:

1. to investigate the difference in Clinical Trials Unit (CTU) related resources that comes from using an adaptive design through a mock costing exercise, compared to non-adaptive designs;
2. to determine reasons for this difference;
3. to propose best-practice guidelines for helping CTUs to ensure adaptive designs are resourced appropriately.

Mock costing exercise

There are five scenarios provided, each inspired by an actual clinical trial. We have made some changes to each trial to avoid comparisons with the actual study. In each scenario a *non-adaptive* and *adaptive* design is proposed. Any differences between these are described in the non-adaptive design and adaptive design sections.

For each scenario, we would like you to provide the resources and their associated costs that would be required for your CTU to support the non-adaptive and adaptive study. A spreadsheet is provided which gives categories to consider. A tab is included for the non-adaptive and adaptive study. Please fill in the two sheets in the spreadsheet, saving a different spreadsheet for each scenario that you cost (see below).

As part of this please also provide resource and costs for the (estimated) work required prior to submission of the grant proposal. This will be useful information for the research regardless of whether you would typically seek to reclaim these costs in the actual grant.

Guidance

1. The scenario documents should provide sufficient detail regarding the planned timelines and workload that the Clinical Trials Unit would require. If you don’t believe there is sufficient data please email [nina.wilson@newcastle.ac.uk](mailto:nina.wilson@newcastle.ac.uk) with the additional information required and we will provide clarifying details. Organising a telephone or skype call will be possible if this would be beneficial.
2. Different CTUs receive different amounts of core-funding and institutional support. Please make every effort to include all resources and costs required for supporting the trial, even if this would not be included in the grant proposal due to being covered by core-funding.
3. Please provide costs at the 100% rate, and including VAT where applicable (e.g. randomisation system provided by a third party).
4. Some CTUs have their own internal statisticians and others work with a separate statistical group within the same institution. If the latter, please ask whoever would provide the statistical costing to take a look too as statistical costs are a specific part of this research.
5. We realise that different CTUs may have different areas of speciality (e.g. a specific therapeutic area). It would be appreciated if as many scenarios were resourced/costed as possible in order to provide the maximal amount of information; however if you would not support a particular type of trial design (e.g. phase IIb dose-ranging) then it is not necessary to provide resources for these scenarios.
6. We have decided not to consider research costs that do not fall within costs/resources required to support the delivery of the trial from the CTU perspective . Please do not include drug/treatment costs or per-patient costs.

Frequently asked questions

Q: Will my costings be seen by anyone else?

A: The only people who will see detailed costings linked to a named CTU will be the Newcastle team (Helen Hancock, Rebecca Maier, James Wason, Nina Wilson) and the Sheffield qualitative researchers who will conduct interviews with you to explore the costings. Otherwise all costs and resources will be anonymised through allocating a code to each CTU.

Q: Will you be comparing costs between CTUs?

A: The primary aim of the study is to determine what the additional resources required for a CTU to effectively support an adaptive clinical trial. Comparing resources between CTUs will be included as part of the analysis along with comparing differences (adaptive vs non-adaptive) between CTUs. The aim is to inform investigation of reasons for differences and subsequent best-practice guidelines. We do not plan to present in depth data at the individual CTU level, but may indicate ranges to underpin our findings.

Q: Should I include costs for health economics?

A: Although estimating any increase in resources/costs for a health economics evaluation of an adaptive design is of interest, we took the decision to not include this as part of the costing exercise. This is because to our knowledge it is rarer for health economics groups to be part of a CTU. If this is different for your CTU and you would be willing, please do add health economic costs as separate rows (this will be removed from the primary comparison but depending on response will potentially be included in a secondary analysis).

Q: What will happen after I provide resource estimates?

A: After all CTUs have provided costs (or by 3^rd^ March) the Newcastle team will summarise the costings into a report for discussion with the group of co-investigators on this project. The report will summarise the different types of costs, comparing between adaptive and non-adaptive equivalents and between scenarios. Due to the low number of data points, this analysis will be descriptive only. After the subsequent meeting of co-investigators we will arrange a mutually beneficial time for a skype interview where we will explore the costing and influences on it. You will be invited to a meeting in Sheffield in late June/early July to review the (anonymised) findings from the qualitative research and to develop best-practice guidelines.
